# Factors influencing in-hospital death for pediatric patients with isolated methylmalonic acidemia: a nationwide inpatient database analysis

**DOI:** 10.1186/s13023-020-01446-0

**Published:** 2020-06-19

**Authors:** Yi-Zhou Jiang, Yu Shi, Ying Shi, Lan-Xia Gan, Yuan-Yuan Kong, Li-Ying Sun, Hai-Bo Wang, Zhi-Jun Zhu

**Affiliations:** 1 National Clinical Research Centre for Digestive Diseases, Beijing Friendship Hospital, Capital Medical University, 95# Yong-an Road, Xi Cheng District, Beijing, 100050 China; 2 China Standard Medical Information Research Centre, Shenzhen, Guangdong China; 3 Clinical Trial Unit, Precision Medicine Institute, First Affiliated Hospital of Sun Yat-Sen University, No.58, Zhong Shan Er Lu, Guangzhou, 510080 China

**Keywords:** Hospitalized, In-hospital, Isolated methylmalonic acidemia, Mortality, Pediatric

## Abstract

**Background:**

Patients with isolated methylmalonic acidemia (MMA) usually experience recurrent episodes of acute metabolic decompensation or metabolic stroke, require frequent hospitalization, and have a relatively high mortality rate. The aim of our study was to assess factors predicting the in-hospital death of pediatric patients with isolated MMA. We performed a retrospective study using data from the Hospital Quality Monitoring System, a national inpatient database in China collected from 2013 to 2017. All patients under 18 years old with a diagnosis of isolated MMA were included. Demographic, hospital-related, and clinical features were collected. Poisson regression was performed to identify potential influencing variables associated with in-hospital death.

**Results:**

From 2013 to 2017, among 2317 admissions for pediatric patients diagnosed with isolated MMA, 1.77% had the outcome of death. In the univariate analysis, patients aged under 1 year had a higher risk of death than did those aged 1 year or older (odds ratio [OR] = 2.63, 95% confidence interval [CI]: 1.36–5.07). There was a higher risk of in-hospital death for patients admitted through emergency departments or via referrals than for those admitted through other routes (OR = 3.76, 95% CI: 1.84–7.67). Deaths were higher in hospitals with volumes of less than 50 patients with isolated MMA during the five study years (OR = 2.92, 95% CI: 1.46–5.83). Moreover, the risk of in-hospital death gradually decreased over time (OR = 0.72, 95% CI: 0.57–0.90). In the multivariate analysis, the abovementioned associations with the risk of in-hospital death remained statistically significant. However, no significant associations were observed between specific clinical signs and in-hospital death in either the univariate or the multivariate analysis.

**Conclusions:**

Younger age, admission to hospitals with low patient volumes, and admission through emergency departments or referrals are associated with higher risk of in-hospital death. The co-existence of specific clinical signs appears to have no effect on in-hospital death.

## Introduction

Methylmalonic acidemia (MMA) is a rare autosomal recessive disorder of propionic acid metabolism induced by the deficiency of methylmalonyl-CoA mutase or in the synthesis of its cofactor adenosylcobalamin [1]. This disease is characterized by an elevated concentration of methylmalonic acid in the patient’s blood and urine, which results from the failure to convert methylmalonyl-CoA into succinyl-CoA during propionyl-CoA metabolism. When this kind of metabolism problem presents without hyperhomocysteinemia or homocystinuria, the disease is called isolated MMA [2].

Patients with isolated MMA typically develop symptoms in the neonatal period, and the clinical features are diverse, usually characterized by metabolic acidosis, hyperammonemia, and even coma. Most patients experience recurrent episodes of acute metabolic decompensation (characterized by metabolic acidosis, hyperammonemia and progressive encephalopathy [3]), requiring frequent hospitalization. In severe cases, the acute metabolic crisis can even lead to death [3–6]. The long-term disease-related complications also include failure to thrive, variable neurocognitive and neurologic deficits, and renal dysfunction [2, 3, 7–11]. Neurological symptoms including seizures/epilepsy, encephalopathy, and renal damage are the most common and dangerous clinical manifestations of isolated MMA occurring in the hospital. The overall prognosis of this disorder is unpromising if it is not promptly treated, with a mortality rate of about 26–40% in patients with isolated MMA [7, 8, 10, 12–15].

MMA was first reported in mainland China in 2000 [16], and hospital admissions for MMA in China have been increasing in recent years. We have previously reported an increasing trend in the proportion of admissions for patients diagnosed with MMA from 2013 to 2017 [17]. To date, most studies on MMA around the world have been single-center studies or case reports with limited sample sizes [8, 18–21]. Moreover, the impact of potential influencing factors on the prognosis of isolated MMA during hospitalization has not yet been explored. Therefore, we conducted a retrospective analysis using a national database to assess the associations between sociodemographic characteristics, specific clinical signs, and in-hospital death among pediatric patients with isolated MMA in China.

## Methods

### Data source

This retrospective study used data from the Hospital Quality Monitoring System (HQMS) [22], a mandatory patient-level registration database of the standardized electronic inpatient discharge records of secondary and tertiary hospitals in China under the administration of the Bureau of Medical Administration and Medical Service Supervision, National Health and Family Planning Commission of the People’s Republic of China. The automatic collection of electronic discharge records by the HQMS began on January 1, 2013.

At each hospital, certified professional medical coders coded the diagnoses stated on the front page of these records using the *International Classification of Diseases, revision 10* (ICD-10) coding system. The front page of each hospitalization record lists one primary/principal diagnosis for each hospitalization and up to 10 secondary diagnoses (including diseases or clinical features that are not the chief reason for the hospitalization), as well as a maximum of three additional pathological diagnoses when a biopsy was performed. When the electronic discharge records are collected from each participating hospital, data-quality control is performed automatically by the system to ensure completeness, consistency, and accuracy.

### Definition of the study population

We included all patients aged under 18 years with any discharge diagnosis of MMA from January 1, 2013, to December 31, 2017, using the ICD-10 code for MMA (E71.102). To identify patients with isolated MMA, we extracted all entries without hyperhomocysteinemia or homocystinuria (E72.101, E72.102 or E72.100 × 007) among the population of pediatric patients with MMA.

### Definition of variables

Sociodemographic details including age, gender, year of discharge, and admission type were extracted from the front page of the hospitalization medical records. Hospital volume was also calculated on the basis of the total number of discharges with a diagnosis of isolated MMA from a specific hospital from 2013 to 2017. The continuous variables (age and hospital volume) were converted to categorical variables by their median values.

Queries were conducted on the patient discharge records to search for specific clinical signs that are recognized as common in isolated MMA [2], filtered through ICD-10 codes. Specifically, the examined clinical signs were seizures/epilepsy (F06.801, F44.500, G40.101, G40.103, G40.309, G40.400, G40.401, G40.404, G40.500, G40.800, G40.800 × 004, G40.804, G40.900, G41.900, P90.× 00, R56.800, R56.801, R56.802, or R56.803), encephalopathy (G80.800, G80.900, G92.× 00, G40.402, G93.400, G93.402, G93.403, P91.900, F07.900, or P91.600), and renal damage (N17.800, N17.900, N18.801, N18.802, N18.803, N18.804, N18.900, N19.× 00, and N18.001). Clinical features were considered present if they were found in either the primary or second secondary diagnosis in the records. The outcome of interest in the study was in-hospital death.

Data collection and analysis were performed according to the ethical standards of the Helsinki Declaration. The study was approved by the Ethical Committee of Beijing Friendship Hospital, Capital Medical University (Approval ID: 2019-P2–154-01).

### Statistical analyses

Continuous variables with highly skewed distributions, including age, are presented using the median value and the interquartile range (IQR). Categorical variables are expressed as frequencies and percentages. Because of the low incidence of in-hospital death, univariate and multivariate Poisson regression was used to examine the potential influencing variables for this outcome. Statistical significance was defined as a two-tailed *P*-value < 0.05. All statistical analyses were performed using SAS software, Version 9.4 (SAS Institute Inc., Cary, NC, United States). The forest plot was depicted using the forestplot package of R, Version 3.5.0.

## Results

### Sociodemographic characteristics of hospitalized pediatric patients with isolated MMA

During the 5-year period analyzed in this study, 2317 isolated MMA pediatric admissions without missing data were identified. As shown in Table 1, the median age was 1 year, and the IQR of age was from 0.25 to 2.00 years old. Over half of these pediatric admissions (*n* = 1337, 57.30%) were male. The majority of cases were routine admissions. Over half of admissions (51.70%) were treated at hospitals with a patient volume of more than 50 during the 5-year study period. The percentage of patients with the outcome of death among all admissions was 1.77% (*n* = 41).

**Table 1:** Sociodemographic characteristics of hospitalized pediatric patients with isolated MMA

Variables	Admissions (***N*** = 2317)
Age	1.00 (0.25–2.00)
Sex	
Male	1337 (57.70%)
Female	980 (42.30%)
Type of admission	
Routine	1357 (58.57%)
Emergency department or referral	788 (34.01%)
Other channels	172 (7.42%)
Hospital volumes in 5 years	
> =50	1198 (51.70%)
< 50	1119 (48.30%)
Year	
2013	241 (10.40%)
2014	382 (16.49%)
2015	471 (20.33%)
2016	589 (25.42%)
2017	634 (27.36%)
In-hospital death	
Yes	41 (1.77%)
No	2276 (98.23%)

### Clinical features of hospitalized pediatric patients with isolated MMA

A small proportion of pediatric patients with isolated MMA had severe clinical symptoms during hospitalization. Seizures/epilepsy was observed in 13.55% of the admissions (Table 2). Encephalopathy occurred in 6.34% of the pediatric patients. There was a relatively low incidence of renal damage (1.86%) in the patients with isolated MMA.

**Table 2:** Severe clinical symptoms of admitted pediatric patients with isolated MMA

Clinical symptoms	N (%)
Seizures/epilepsy	
Yes	314 (13.55%)
No	2003 (86.45%)
Encephalopathy	
Yes	147 (6.34%)
No	2170 (93.66%)
Renal damage (Acute/chronic)	
Yes	43 (1.86%)
No	2274 (98.14%)

### Univariate and multivariate analyses of sociodemographic factors

**Fig. 1:**
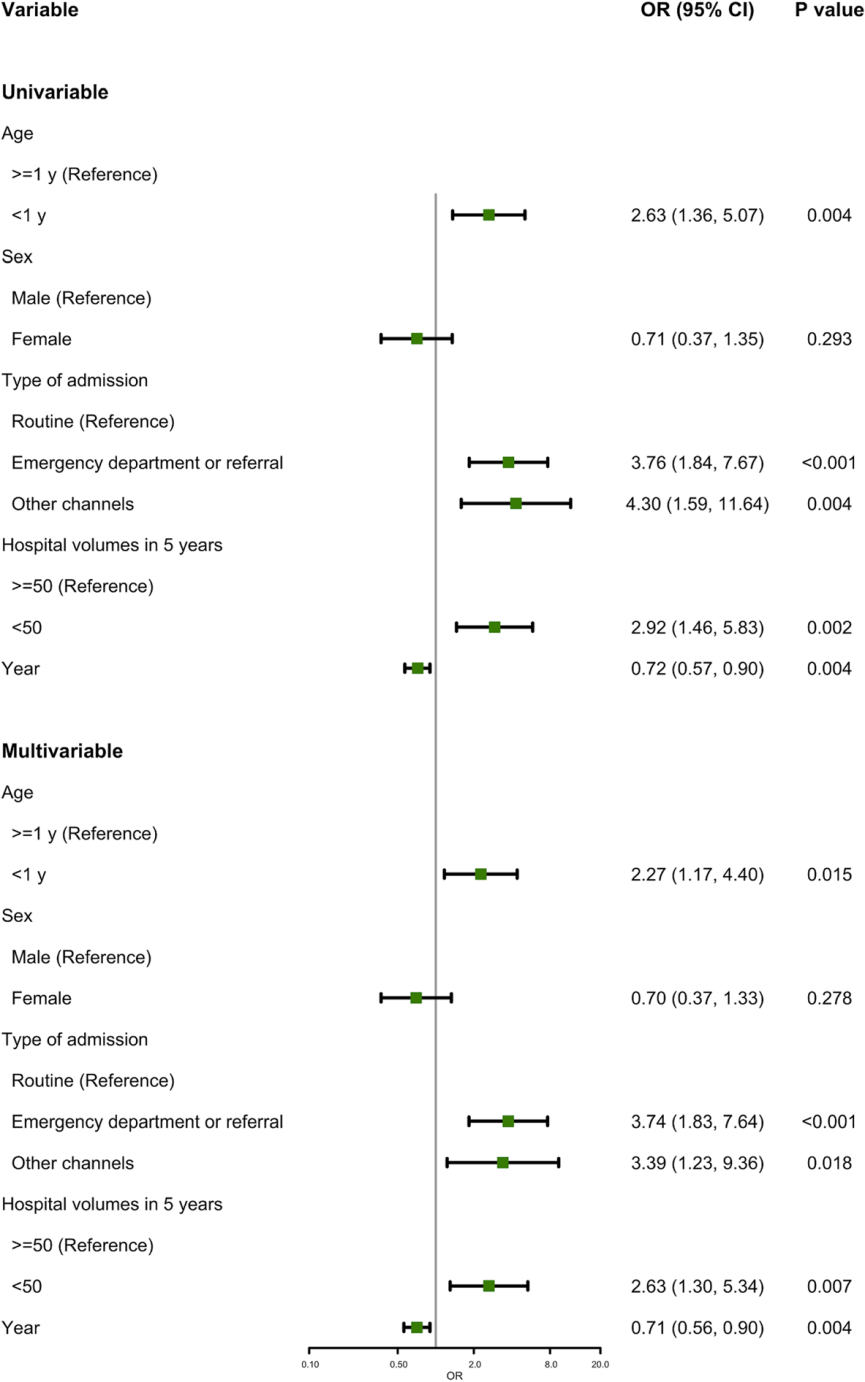
The effects of the potentially influencing factors on in-hospital death

In the univariate analysis, pediatric patients aged under 1 year had higher mortality than did those aged 1 year or older (odds ratio [OR] = 2.63, 95% confidence interval [CI]: 1.36–5.07). Compared with routine admissions, patients admitted through emergency departments or referrals (OR = 3.76, 95% CI: 1.84–7.67) or through other channels (OR = 4.30, 95% CI: 1.59–11.64) had higher risk of in-hospital death. The pediatric patients in the hospitals with patient volumes of less than 50 had a higher risk of death (OR = 2.92, 95% CI: 1.46–5.83). The risk of in-hospital death gradually decreased over time (OR = 0.72, 95% CI: 0.57–0.90). No statistical difference in the risk of death was observed between boys and girls (*P* = 0.293). In the multivariate analysis, age, type of admission, hospital’s total admissions of patients with isolated MMA, and year of admission remained statistically significantly associated with the risk of in-hospital death (Fig. 1).

### Univariate and multivariate analyses of clinical features

We assessed the associations of in-hospital death with three clinical features during hospitalization: seizures/epilepsy, encephalopathy, and renal damage. As shown in Table 3, in the univariate and multivariate analyses, pediatric patients with any one of these clinical signs were not at statistically higher risk of in-hospital death than were those with none of these signs.

**Table 3:** Clinical features influencing mortality by univariate and multivariate Poisson regression analysis in pediatric patients with isolated MMA (*N* = 2317)

	Univariate			Multivariate		
	OR	95% CI	*P*	OR	95% CI	*P*
Seizures/epilepsy						
No	Reference			Reference		
Yes	0.69	(0.25, 1.93)	0.480	0.69	(0.25, 1.95)	0.490
Encephalopathy						
No	Reference			Reference		
Yes	1.17	(0.36, 3.78)	0.799	1.15	(0.35, 3.72)	0.819
Renal damage (Acute/Chronic)						
No	Reference			Reference		
Yes	1.32	(0.18, 9.62)	0.783	1.27	(0.17, 9.23)	0.816

## Discussion

Isolated MMA, an inherited metabolic disorder, is among the most common organic acidemias worldwide. The present study included 2317 hospitalizations of pediatric patients with isolated MMA in China from 2013 to 2017. This study identified age, type of admission, year of hospitalization, and hospital volume as potential factors associated with the risk of death in hospitalized patients with isolated MMA.

Previous work has reported that the overall outcome for patients with isolated MMA remains poor, despite the existence of apparently effective medications and low-protein diets [7, 12, 13]. A previous study, conducted in 2013, reported a mortality rate of 26.7% among 30 cases of isolated MMA in China with regular follow-ups with an age range of 1 month to 8 years [15]. This high mortality rate may be related to insufficient recognition of the disease and delayed diagnosis because of nonspecific clinical presentation and a lack of awareness among physicians because of the rarity of MMA. In 2014, on the basis of an evaluation of all existing evidence and expert group meetings, recommendations about the management of MMA were put forward [3]. These guidelines provided a consensus on common standards of care, which facilitates the dissemination of good practices and enables improvements in therapeutic effectiveness. The prognosis of isolated MMA has improved over time [13]. We also found a decreasing trend in in-hospital death among patients over time, which may suggest the effectiveness of the abovementioned guidelines. As the most common inherited metabolic disorder seen in neonatal intensive care units in China [23], MMA has attracted much concern. Another plausible explanation for the decline in mortality over time is that early diagnosis because of the wider application of screening tools, namely tandem mass spectrometry and gas chromatography/mass spectrometry, could result in early management and treatment. This advancement, in turn, may have improved the prognosis of pediatric patients.

We found that being aged less than 1 year was a risk factor for in-hospital death among patients with isolated MMA, which may be explained by the natural history of the disease. Catastrophic neonatal presentations of isolated MMA develop rapidly from vomiting to anorexia, coma, and death. Even invasive therapy cannot prevent the progression of the disease. This is consistent with a previous study showing that survival was strongly influenced by age at onset, and onset in newborns is an independent negative risk factor for survival among patients with MMA [15, 19, 21]. Most cases in the present study were routine admissions rather than admissions via emergency departments or referrals. In China, when outpatients in relatively poor condition require further systematic examination or comprehensive treatment, they are admitted to the inpatient ward (i.e. through routine admission). In our study, routine admission was associated with a lower death risk compared with admission via emergency departments or referrals. It is likely that symptoms of patients with isolated MMA admitted through emergency departments or other health care settings tended to be more serious than those of patients with routine admission. The clinical manifestations of patients with isolated MMA are complex, making it likely for patients to present with metabolic crisis, which is potentially fatal [3]. All reported triggers of acute decompensation, such as infection, fever, surgery, and acute trauma, may lead to emergency situations for pediatric patients. Although the results showed that boys outnumbered girls among the isolated MMA admissions, there was no significant difference in the risk of in-hospital death by sex.

Previous studies have shown that higher hospital volume is associated with better outcomes for patients undergoing surgical procedures and for those with acute liver failure [24–28]. However, there has been limited information regarding this relationship for rare diseases such as MMA. Our study demonstrated that hospitals with more experience in treating patients with isolated MMA had a lower death risk in comparison with hospitals that had treated fewer of these patients. For patients with MMA who are critically ill, bicarbonate correction, carnitine administration, a variety of antibiotic regimens, specific protein intake, regular monitoring of blood gases, supportive care for acute decompensation, and other fluid and nutritional management all need to be considered under careful evaluation [3]. Therefore, the management of patients with isolated MMA requires support from multidisciplinary and specialized teams. This suggests that hospitals with more experience in treating isolated MMA are more likely to provide more comprehensive care, as well as early recognition and definite diagnosis. Additionally, liver transplantation, which has been shown to correct metabolic instability [29–31], may be more likely to be available in these hospitals.

Because of the deficiency of a specific enzyme, isolated MMA is always associated with progressive multiple organ damage. In addition to metabolic acidosis, there are other clinical signs such as seizures/epilepsy and encephalopathy, which suggest the involvement of the nervous system, and these can sometimes even lead to coma. According to a previous study, acute or chronic renal damage is attributed to tubulointerstitial nephritis with progressive renal failure [3]. Notably, our study demonstrated that the risk of in-hospital death was not statistically higher for pediatric patients with any one of the examined clinical features than for other patients. We postulate several reasons for this finding. The occurrence of specific signs often indicates the need for the intensification of therapeutic regimens in the hospital, which can prevent further deterioration and serious complications. For some patients with predictable poor prognoses, if these measures are available, liver and/or kidney transplantation can be considered by doctors during hospitalization. It may also be the case that patients with severe complications are more likely to be managed in relatively high-level hospitals, which leads to better outcomes.

Our study had several limitations. First, the HQMS dataset was unable to cover all secondary and tertiary hospitals in China. Further, we relied entirely on ICD-10 codes for diagnosis and case selection. It was difficult to distinguish between pre-existing and new-onset clinical features, between previously established diagnosis and first-time diagnosis, which precluded further analysis. Furthermore, MMA subtype, such as mut0 enzymatic or cblB, has been reported to be associated with mortality [32, 33], but this information was unavailable in the dataset. Other clinical data such as laboratory features and imaging results, which might indicate the risk of death, were also not accessible.

Despite the above limitations, our study also had several advantages, including a relatively large sample size consisting of pediatric patients with isolated MMA from a nationwide database. This study also revealed some general information, including the demographic characteristics and incidences of specific clinical features among hospitalized pediatric patients with isolated MMA in China. Moreover, we further explored the associations between potentially influential factors and in-hospital death. The information presented in this article will help in the identification of specific factors that might have the potential to improve the prognosis of isolated MMA.

## Conclusions

Some pediatric patients with isolated MMA experienced severe signs including seizures/epilepsy, encephalopathy, and renal damage. However, the appearance of these symptoms may not impact the risk of in-hospital death. Younger age of pediatric patients, lower hospital volume, and admission through emergency departments or referrals are associated with a higher risk of in-hospital death.

## Data Availability

The datasets generated and analyzed during the current study are available from the corresponding author on reasonable request.
